# Characteristics and outcomes of COVID-19 patients during the BA.5 omicron wave in Tehran, Iran: a prospective observational study

**DOI:** 10.1186/s12879-023-08181-4

**Published:** 2023-04-17

**Authors:** Mohammadreza Salehi, Arezoo Salami Khaneshan, Abbas Shakoori Farahani, Mahsa Doomanlou, Mohammad Arabzadeh, Abolfazl Sobati, Kousha Farhadi, Reza Fattahi, Esmaeil Mohammadnejad, Asghar Abdoli, Jayran Zebardast

**Affiliations:** 1 Research center for antibiotic stewardship and antimicrobial resistance, Infectious diseases department, Imam Khomeini Hospital Complex, Tehran University of Medical Sciences, Tehran, Iran; 2 Infectious diseases department, Imam Khomeini Hospital Complex, Tehran University of Medical Sciences, Tehran, Iran; 3 Department of Medical Genetics, School of Medicine, Imam Khomeini Hospital Complex, Tehran University of Medical Sciences, Tehran, Iran; 4 Molecular Genetic Ward, Imam Khomeini Hospital Complex, Tehran University of Medical Sciences, Tehran, Iran; 5 COVID-19 laboratory, Imam Khomeini Hospital Complex, Tehran University of Medical Sciences, Tehran, Iran; 6 Department of Nursing and Midwifery, Imam Khomeini Hospital Complex Tehran University of Medical Sciences, Tehran, Iran; 7 Research center for antibiotic stewardship and antimicrobial resistance, Department of Medical- Surgical Nursing and Basic Sciences, School of Nursing and Midwifery, Tehran University of Medical Sciences, Tehran, Iran; 8 Department of Hepatitis and AIDS, Pasteur Institute of Iran, Tehran, Iran; 9 Advanced Diagnostic and Interventional Radiology Research Center (ADIR), Tehran University of Medical Science, Tehran, Iran

**Keywords:** Clinical characteristics, Iran, Omicron BA.5, COVID-19

## Abstract

**Background:**

Omicron (B.1.1.529) is the fifth variant of concern of SARS-CoV-2, which has several subvariants. Clinical features of BA.1 and BA.2 infections have been described in the literature, but we have limited information about the clinical profile of BA.5, which caused the seventh wave in Iran.

**Methods:**

A prospective observational study was conducted on the BA.5 confirmed patients referred to Imam Khomeini Hospital Complex, Tehran, Iran, from 11th to 31st August 2022. The patients were divided into the two groups of outpatients and hospitalized patients, and their clinical, radiological, and laboratory data and outcomes were recorded and analyzed.

**Results:**

We included 193 patients with confirmed BA.5 infection, of whom 48 patients (24·8%) were hospitalized. The mean age of the patients was 45·3 ± 16·5 years, and 113 patients (58·5%) were female. The mean number of days patients had symptoms was 6·8 ± 2·4 days. The most common symptoms were weakness (69·9%), sore throat (67·4%), myalgia (66·3%), hoarseness (63·7%), headache (55·4%), fatigue (54·9%), and dry cough (50·3%). Fever and dyspnea were significantly more observed in the hospitalized patients (p < 0·0001). The COVID-19 vaccination rate was significantly lower in hospitalized patients than in outpatients (35/48–72·9% vs. 140/145 − 96·6%, p < 0·0001). The most common underlying diseases were hypertension (16·1%), diabetes mellitus (9·8%), and cardiovascular diseases (9·8%), all of which were significantly more common in hospitalized patients. Lung opacities were observed in 81·2% of hospitalized patients. By the end of our study, 1·5% of patients died despite receiving critical care services.

**Conclusions:**

Our findings suggested that BA.5 symptoms are more non-respiratory and usually improve within 7 days. Although the proportion of hospitalized patients is still significant, very few patients require intensive care. COVID-19 vaccination is effective in reducing the hospitalization rate.

**Trial registration:**

Not applicable. This study is not a clinical trial.

## Background

The Omicron (B.1.1.529) variant is the fifth variant of concern of SARS-CoV-2, which was first introduced in South Africa on November 25, 2021 and within a few days, caused a new wave of SARS-CoV-2 in South Africa and several other countries around the world [1, 2]. In South Africa, a higher and faster wave peak with less hospitalization, less severe illness, and ultimately fewer deaths than the previous waves were seen [3]. The reports from other countries also showed a reduced risk of severe disease in patients with the Omicron variant compared with the Delta variant [4–7]. The biological evidence suggests that Omicron has a lower virulence than the previous variants due to having different cellular entry mechanisms and preferential proliferation site in the respiratory tract tissue instead of the lung parenchyma [8]. It is unknown whether the SARS-CoV-2 previous infection and/or COVID-19 vaccination can provide protection against severe Omicron infection [9].

Among the dominant Omicron subvariants, BA.4 and BA.5, first isolated in South Africa in early 2022, are offshoots of Omicron BA.2 (despite BA.2 having fewer mutations than BA.1). These additional mutations in BA.4 and BA.5 seem to have associated them with a greater transmission potential [10, 11].

Previous studies show that patients with Omicron BA.2 were not associated with poorer outcomes than patients with BA.1 [12]. As the BA.5 subvariant emerged and became dominant, some reports showed increased hospitalization and even higher mortality [13–15].

Despite many reports on the characteristics of Omicron, more detailed epidemiological and clinical studies are required on this dominant variant and its emerging subvariants.[16].

In this study, we investigated the clinical symptoms, radiological features, laboratory characteristics, and outcomes of COVID-19 patients infected with the Omicron BA.5 subvariant in Tehran, Iran.

## Methods

### Study design and setting

A prospective observational study was conducted on the COVID-19 patients referred to Imam Khomeini Hospital Complex, Tehran, Iran, from August 11, 2022, to August 31, 2022, during the seventh wave (Omicron BA.5 wave).

### Participants

All patients ≥ 12 years old with a positive SARS-CoV-2 test and confirmed Omicron BA.5 infection were included in the study. Patients with a positive SARS-CoV-2 test and confirmed Omicron BA.5 subvariant were identified and divided into two groups of hospitalized patients and outpatients.

### SARS-CoV-2 ***variants identification***

First, the nasopharyngeal samples from suspected COVID-19 patients were collected and transported to the hospital laboratory in falcons containing viral transport media (VTM). Then, the RNA extraction was conducted using the automated magnetic bead-based extractor (MagCore ®, RBC Bioscience, Taiwan) or the manual column-based kit (BehGene, Iran), following the virus inactivation in each sample. The extracted samples were placed in the freezer for further amplification and quantification by real-time polymerase chain reaction (RT-PCR) method using different kits, including Genova (USA), HanaGene (Iran), and COVITECH (Iran) and the detection system (CFX96, Bio-Rad, USA). All steps were performed according to the standard protocols of the kits.

### Procedure and outcomes

**Fig. 1:**
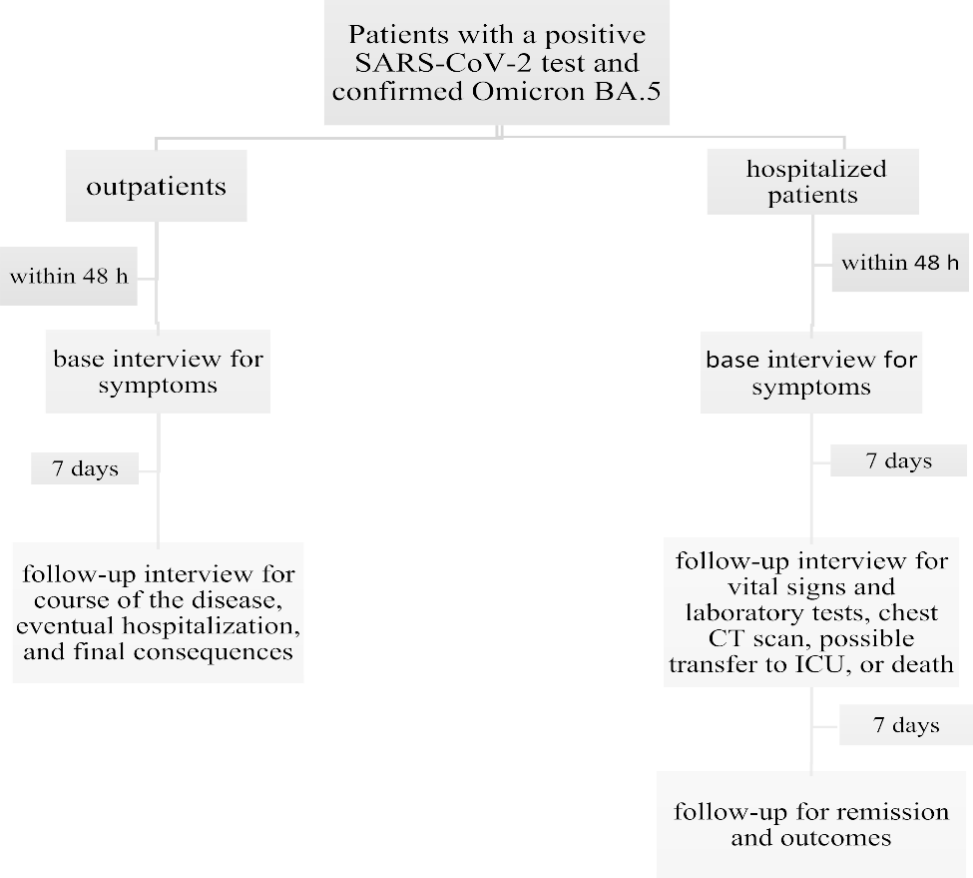
Study design chart

The patients in each group were interviewed in person or by phone within 48 h of testing positive. The clinical information obtained regarding patients’ symptoms and conditions was recorded in data collection forms. The groups were then monitored for any laboratory tests and lung imaging ordered by patients’ clinicians. The research team did not intervene in the clinical and therapeutic management of the patients. The follow-up interview was performed on both groups seven days after the first interview. The outpatients were contacted by phone regarding the course of the disease, eventual hospitalization, and final consequences. Hospitalized patients were interviewed face-to-face regarding their vital signs, laboratory tests, and radiological signs in the chest CT scan. The clinical process and possible transfer to the intensive care unit or death were also recorded. The hospitalized patients were followed for two weeks after the first interview (Fig. 1).

### Data analysis

We used descriptive statistics to analyze the details of clinical features, paraclinical characteristics, clinical course evidence, and death in the BA.5 patients during this time period. All patients who were still hospitalized and did not complete the two-weeks follow-up were excluded from the study. Analysis of laboratory tests was restricted to patients admitted to the hospital. The data were analyzed using the SPSS software version 20.0. The Mean and standard deviation (± SD) were used to display the quantitative data, and the frequency was used to display the qualitative data. The Chi-square analysis was used to compare qualitative variables between the groups of hospitalized and outpatients. The t-test analysis was also used to check the quantitative data between the two groups. An alpha level of less than 5% was considered significant.

## Results

After the evaluation of 207 patients with SARS-CoV-2 RT-PCR positive test, we included 193 patients with confirmed Omicron BA.5 infection in the study (48 (24·8%) hospitalized patients, and 145 (75.2%) outpatients). As seen in Table 1, the mean (± SD) age of the patients was 45·3 (± 16·5) years. One hundred thirteen patients (58·5%) were female, yet a significant proportion of hospitalized patients was male compared with the outpatients (56·3% vs. 36·6%, p < 0·0001). The mean (± SD) number of days patients had symptoms was 6·8 (± 2·4) days. The worst symptoms appeared on the third day (2·5 ± 1·1) from the disease onset in outpatients and on the second day (1·7 ± 0·9) in hospitalized patients (p < 0·0001).

**Table 1:** Baseline characteristics of patients with Omicron BA.5

	Outpatients n = 145	Hospitalized patients n = 48	Total N = 193	P value
**Age** **(mean ± SD)**	40.6 ± 12	63.3 ± 18	45.3 ± 16.5	< 0.0001^a^
**Gender** **n (%)**				
Male	53 (36.6)	27 (56.3)	80 (41.5)	< 0.0001^b^
Female	92 (63.4)	21 (43.8)	113 (58.5)	
**Worst day of symptoms (mean ± SD)**	2.5 ± 1.1	1.7 ± 0.9	2.4 ± 1.2	< 0.0001^a^
**Symptomatic days (mean ± SD)**	5.4 ± 2.3	6.2 ± 1.5	6.8 ± 2.4	0.03^a^
**Underlying condition n (%)**				
Hypertension	15 (10.3)	16 (33.3)	31 (16.1)	< 0.001^b^
Diabetes mellitus	6 (4.1)	13 (27.1)	19 (9.8)	< 0.001^b^
Ischemic heart diseases	5 (3.4)	14 (29.2)	19 (9.8)	< 0.001^b^
Malignant disease	8 (5.5)	11 (22.9)	19 (9.8)	< 0.001^b^
Hypothyroidism	9 (6.2)	4 (8.3)	13 (6.7)	0.740^b^
Chronic lung diseases	4 (2.8)	0 (0.0)	4 (2.1)	0.574^b^
Pregnancy	3 (2.1)	1 (2.1)	4 (2.1)	0.575^b^
None	97 (66.9)	6 (12.5)	29 (14)	
**Vaccination status n (%)**				
Unvaccinated	5 (3.4)	13 (27.1)	18 (9.3)	< 0/0001^b^
**First visit vital signs (mean ± SD)**				
Pulse rate (beats/minute)	94.2 ± 16.7	108.8 ± 19.3	101.9 ± 19.5	< 0.0001^a^
Respiratory rate (breaths/minute)	18.4 ± 5.1	20.4 ± 4.6	19.3 ± 4.9	0.011^a^
Systolic blood pressure (mmHg)	109.4 ± 25.9	123.5 ± 18.9	119.3 ± 2.0	0.018^a^
Temperature (°C)	37.6 ± 0.8	38.0 ± 1.2	37.9 ± 1.1	0.04^a^

^a^ t-test ^b^ chi-square test P < 0.05 was considered significant

**Fig. 2:**
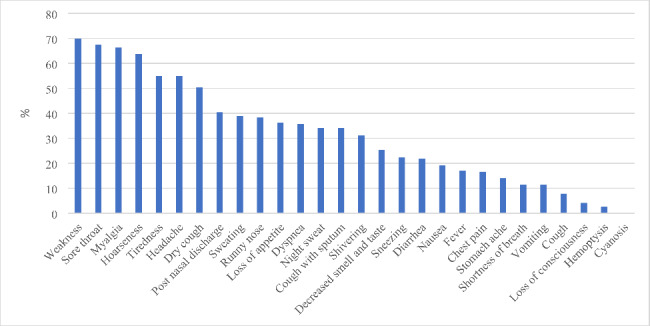
The frequency of baseline clinical symptoms in Patients with Omicron BA.5

**Fig. 3:**
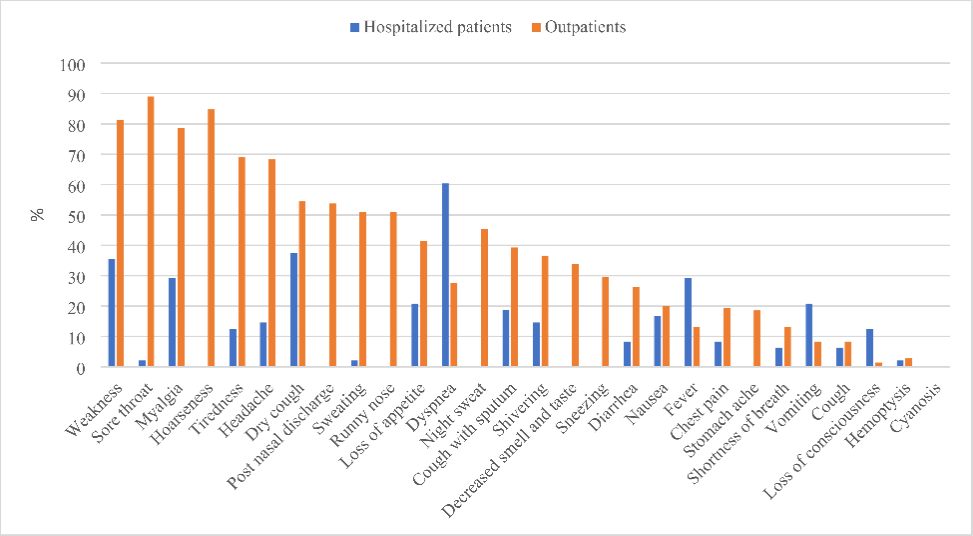
Comparison of baseline clinical symptoms of Omicron BA.5 between outpatients and hospitalized patients

As seen in Fig. 2, the most common baseline symptoms were weakness (69·9%), sore throat (67·4%), myalgia (66·3%), hoarseness (63·7%), headache (55·4%), fatigue (54·9%), and dry cough (50·3%). Dyspnea (27.6% vs. 60.4%, p < 0.0001) and fever (T > 38ºC) (13% vs. 29%, p = 0.01) were significantly more common in hospitalized patients than in outpatients (Fig. 3). None of our patients were asymptomatic.

In terms of the first visit vital signs, the mean (± SD) air room SpO2 was 93·9 (± 4·6%), and the mean (± SD) body temperature was 37·9 (± 1·0) ºC. Fever (T > 38ºc) was significantly more common in hospitalized patients than in outpatients (13% vs. 29%, p = 0·01).There were also significant differences between the two group in pulse rate, respiratory rate, and systolic blood pressure.

The most common underlying diseases were hypertension (16·1%), diabetes mellitus (9·8%), and ischemic heart diseases (9·8%), all of which were significantly more common in hospitalized patients (hypertension: 33·3%, ischemic heart diseases: 29.2%, diabetes mellitus: 27·1%) than in outpatients (87·5% vs. 33·1%, p < 0·0001).

**Fig. 4:**
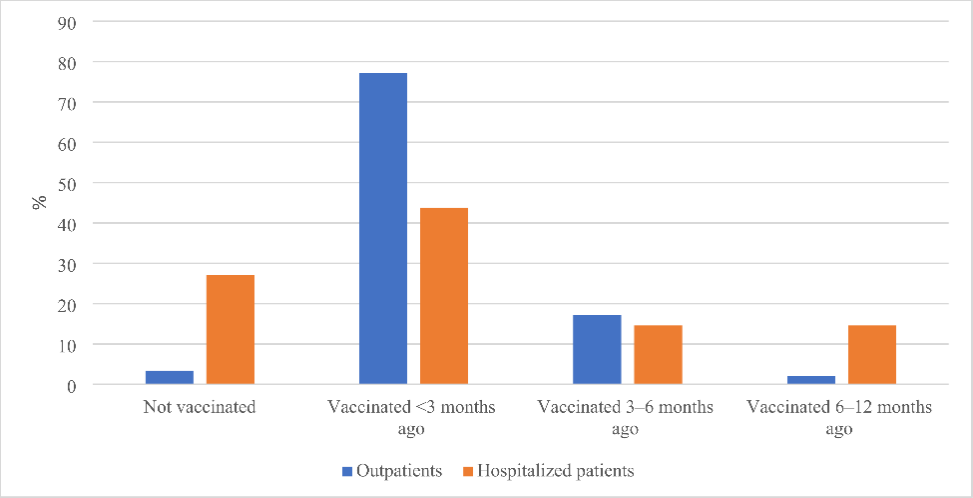
Comparison of the last dose receiving time of COVID-19 vaccine between outpatients and hospitalized patients with Omicron BA.5

Eighteen patients (9·3%) were unvaccinated for COVID-19, and the vaccination rate was significantly lower in hospitalized patients than in outpatients (35/48–72·9% vs. 140/145 − 96·6%, p < 0·0001). As seen in Fig. 4, of 133 patients receiving the last dose of the COVID-19 vaccine within three months before the BA.5 infection, 84·2% were outpatients and 15·8% were hospitalized, while of 10 patients receiving the last dose more than six months ago, 70% needed hospitalization (p < 0·0001).

The results of paraclinical tests (Table 2), which were performed only for hospitalized patients, showed a high level of CRP (79·8 ± 56.8 mg/L). Among the 25·4% of patients who underwent CT scanning, lung opacity was observed in 39 patients (81·2%), and ground glass opacity was the most common pattern seen in 19 patients (39·6%).

**Table 2:** Baseline paraclinical features of hospitalized patients with BA.5 Omicron

**Blood cell count (mean ± SD)**	
White blood cells (×10^9^/mL)	10.4 ± 7.2
Lymphocyte (×10^9^/mL)	2.0 ± 1.7
Neutrophil (×10^9^/mL)	7.6 ± 1.7
Platelet (×10^9^/mL)	195.6 ± 101.8
Hemoglobin (g/dL)	11.4 ± 3.0
**Inflammatory markers (mean ± SD)**	
Lactate dehydrogenase (IU/L)	481.0 ± 160.0
Procalcitonin (ng/mL)	0.5 ± 0.5
C-reactive protein (mg/dL)	79.8 ± 56.7
Erythrocyte sedimentation rate (mm/h)	50.2 ± 30.3
D-dimer (ng/mL)	3181.2 ± 2914.3
**Chest CT scan (n (%)**	
Normal	8 (17.8%)
Ground-glass opacity	19 (39.6%)
Consolidation	3 (6.3%)
Nodule	2 (4.2%)
Reticular pattern	2 (4.2%)
Mixed pattern	16 (33.3%)
Bilateral involvement	22 (45.8%)
< 25% involvement	9 (18.8%)
25–50% involvement	25 (52.1%)
50% < involvement	15 (31.3%)

In terms of the treatment regimen, 35 hospitalized patients (72·9%) were treated with remdesivir and 29 (60·4%) received dexamethasone. Outpatients did not receive any antiviral agents.

At the end of the study (day 14 for hospitalized patients), 13 patients (6·7%) were still hospitalized (none of them were in the ICU), and 3 hospitalized patients (1·5%) died despite receiving critical care services including mechanical ventilation.

## Discussion

We examined the clinical features and outcomes of SARS-CoV-2 infection among Iranian individuals with the Omicron BA.5 subvariant.

Although the average age of our patients was 45·3 years and none were asymptomatic, in a study on patients with BA.1 subvariant in China, the mean age was 34·5 years and 25% were asymptomatic [17]. In a review on Omicron BA.2 clinical studies, 58·1% of patients were found asymptomatic and only 4·2% experienced severe symptoms [18]. Our results suggest that the rate of symptomatic patients is probably higher in BA.5 infection than in previous Omicron subvariants infections.

Our findings also suggest that non-respiratory symptoms were the most common symptoms in patients with BA.5 Omicron infection, unlike in patients with COVID-19 caused by previous variants in whom respiratory symptoms were the most frequent symptoms [19, 20]. Similarly, in a study by Menni et al. on British patients with Omicron in late 2021, the most common clinical features were non-respiratory symptoms [21]. However, some symptoms such as weakness, myalgia, and hoarseness were more common in our study, sore throat was almost equally common in both studies, and sneezing and runny nose were clearly less common in our findings [21].

The most common symptom of patients with BA.5 Omicron subvariant in our study was weakness, and fever was less common (17·1%) than in a previous BA.1 Omicron report (32·8%). [17] However, fever was the most common symptom (> 80%) in early reports of the COVID-19 pandemic [19, 22, 23]. In our results, similar to the early COVID-19 results, cough was the most common respiratory symptom, and it was significantly more common in hospitalized patients with BA.5 Omicron [24].

The mean symptomatic period in patients with BA.5 infection in our study was about 7 days, which is consistent with the symptomatic days of early Omicron patients. This period was ten days in the reports before the Omicron wave [21, 25, 26]. It seems that Omicron and its subvariants cause a shorter symptomatic period than the previous SARS-CoV-2 variants. In previous studies, the most common underlying diseases associated with COVID-19 were hypertension, diabetes mellitus, and cardiovascular diseases, which were reported in the same order in our study [23, 27–29]. Hypertension and diabetes mellitus were similarly reported as the most common underlying diseases in patients with the BA.1 Omicron subvariant [17].

The rate of ICU admission and death in BA.5 hospitalized patients, which was reported low in our study and among individuals infected with earlier Omicron subvariants, is significantly lower than that in patients with pre-Omicron variants infections based on several large-scale studies [12, 30, 31].

The higher hospitalization rate among unvaccinated patients seen in our study confirms the COVID-19 vaccine effectiveness in reducing the risk of severe disease caused by any virus variant, including Omicron.[32–34]. The lower admission rate in patients during the Omicron wave can be explained by the combination of the lower virulence of Omicron and the immunity acquired from both vaccination and previous infections, so-called hybrid immunity [3, 35].

In terms of inflammatory markers, the CRP value was high in our BA.5 hospitalized patients, like in patients with other variants of SARS-CoV-2 [36–38]. However, compared with the reported CRP value for BA.1 and BA.2 in previous studies, the mean CRP level was higher in our study [17, 18]. The CRP level was reported to be significantly higher in patients with the Delta variant [19, 38].

Similar to reports from previous Omicron subvariants, ground glass opacity and bilateral involvement were the most common results of CT scan in our study [17, 39, 40].

The mortality rate in our BA.5 patients (1·5%), which was not associated with vaccination or any specific risk factor, seems to be lower than that in patients infected with the wild-type variant [19, 41, 42] and previous Omicron variants [5].

We were able to obtain useful clinical information about Omicron BA.5. However, since the data used in this study collected only from one hospital, the mortality group was too small to allow performing subgroup analyses. Another limitation of this study was the lack of sufficient information about the patients’ history of previous COVID-19 infections and its possible immunological effects on the symptoms, severity, and outcome of the current disease. There were also no laboratory results for outpatients because according to the national protocol for COVID-19 management, laboratory tests are requested only for hospitalized patients.

## Conclusions

Our Findings suggest that BA.5 symptoms are more non-respiratory and usually improve within seven days. Although the proportion of hospitalized patients is still significant, very few patients require ICU admission. COVID-19 vaccination is effective in reducing the hospitalization rate in patients with BA.5.

## Data Availability

The datasets used and/or analyzed during the current study are available from the corresponding author on reasonable request.

## List of Abbreviations

SARS-CoV-2: Severe acute respiratory syndrome coronavirus 2
COVID-19: Coronavirus disease 2019
CRP: C-reactive protein
CT: Computed tomography
ICU: Intensive care unit
