# Correction to: Characteristics and outcomes of COVID-19 patients during the BA.5 omicron wave in Tehran, Iran: a prospective observational study

**DOI:** 10.1186/s12879-023-08507-2

**Published:** 2023-08-07

**Authors:** Mohammadreza Salehi, Arezoo Salami Khaneshan, Abbas Shakoori Farahani, Mahsa Doomanlou, Mohammad Arabzadeh, Abolfazl Sobati, Kousha Farhadi, Reza Fattahi, Esmaeil Mohammadnejad, Asghar Abdoli, Jayran Zebardast

**Affiliations:** 1 Research center for antibiotic stewardship and antimicrobial resistance, Infectious diseases department, Imam Khomeini Hospital Complex, Tehran University of Medical Sciences, Tehran, Iran; 2 Infectious diseases department, Imam Khomeini Hospital Complex, Tehran University of Medical Sciences, Tehran, Iran; 3 Department of Medical Genetics, School of Medicine, Imam Khomeini Hospital Complex, Tehran University of Medical Sciences, Tehran, Iran; 4 Molecular Genetic Ward, Imam Khomeini Hospital Complex, Tehran University of Medical Sciences, Tehran, Iran; 5 COVID-19 laboratory, Imam Khomeini Hospital Complex, Tehran University of Medical Sciences, Tehran, Iran; 6 Department of Nursing and Midwifery, Imam Khomeini Hospital Complex Tehran University of Medical Sciences, Tehran, Iran; 7 Research center for antibiotic stewardship and antimicrobial resistance, Department of Medical- Surgical Nursing and Basic Sciences, School of Nursing and Midwifery, Tehran University of Medical Sciences, Tehran, Iran; 8 Department of Hepatitis and AIDS, Pasteur Institute of Iran, Tehran, Iran; 9 Advanced Diagnostic and Interventional Radiology Research Center (ADIR), Tehran University of Medical Science, Tehran, Iran

**Correction to**: ***BMC Infect Dis*** **23, 237 (2023).**

10.1186/s12879-023-08181-4

The original publication of this article contained 1 error:

1. The author name “**Mahsa Doomanlu**” should have been “**Mahsa Doomanlou**”.

The original article has been updated to correct this error.

